# Intelligent Patient Appointment Schedules

**DOI:** 10.3390/healthcare14091195

**Published:** 2026-04-29

**Authors:** Salma Elhag, Lama Althagafi, Shroog Almouabdi

**Affiliations:** Information Systems Department, Faculty of Computing & Information Technology, King Abdulaziz University, Jeddah 21589, Saudi Arabia

**Keywords:** intelligent appointment scheduling, medical chatbot, healthcare automation, clinical triage, machine learning, natural language processing (NLP), patient data analysis, Six Sigma approach

## Abstract

**Background**: Hospital appointment systems suffer from extended patient waits, manual interventions, and suboptimal resource allocation, reducing satisfaction and efficiency. **Methods**: This study develops IPAS using Business Process Analysis (BPA), Bizagi modeling for As-Is/To-Be workflows, SWOT analysis, TQM, and Six Sigma DMAIC. It integrates ML/NLP with BioBERT-BiLSTM triage (AUC 0.92, F1 0.87) for symptom analysis, specialist matching, and automated booking, validated via Bizagi simulations. **Results**: Simulations show booking time was reduced 96.3% (155 to 5.73 min) and human intervention was cut 70%, with enhanced patient satisfaction and process capability. **Conclusions**: IPAS demonstrates simulation-based gains in scheduling efficiency, pending real-world validation.

## 1. Introduction

Healthcare institutions achieve better performance through their appointment scheduling systems, which operate with maximum efficiency. The current hospital scheduling systems face multiple operational problems because they lead to extended patient delays and require hands-on resource management and insufficient medical resource optimization [1]. The operational demands on healthcare personnel increase while patient satisfaction levels decrease because of these system problems. The development of artificial intelligence (AI) and data analytics enables intelligent automation to become an effective solution for healthcare process optimization. The intelligent patient appointment system used artificial intelligence to create an automated clinical triage system, which combined with natural language processing for appointment scheduling optimization. The system allows patients to enter their symptoms through chat or upload images, which enables the system to match their information with medical records for specialist recommendations and immediate booking capabilities. The system implemented multiple analytical and quality management techniques to achieve process excellence and ongoing development through Business Process Analysis (BPA), SWOT analysis, Six Sigma (DMAIC), and Total Quality Management (TQM). The Bizagi Modeler tool allowed the team to create models of the current appointment system (As-Is) and the future intelligent system (To-Be) for performance evaluation and KPI assessment of waiting times, operational efficiency, and accuracy levels. The research aims to show how automated systems combined with systematic improvement methods can create better healthcare appointment systems that use data to deliver improved services for patients.

## 2. Literature Review

Healthcare facilities need efficient appointment scheduling systems because each passing minute determines patient results. The implementation of artificial intelligence (AI) and data-driven systems requires healthcare organizations to optimize their appointment management systems. The evaluation of ten research studies demonstrates how modern scheduling systems enhance appointment precision, shorten patient delays, and maximize healthcare facility resource usage. The multi-appointment patient scheduling model combined machine learning with optimization to handle chemotherapy appointment complexities, which include sequential treatment sessions with different resource requirements. The hybrid SADNSOB model used artificial neural networks (ANNs) to forecast patient absences and appointment lengths, which resulted in maximum performance for scheduling appointments between patients and staff members [1]. The research team created an electronic appointment system through AngularJS, AJAX, PHP and MySQL integration to create automated registration and enhance user interface functionality. The system decreased human mistakes and decreased patient delay, but researchers did not test its performance in actual healthcare settings and security measures [2]. The authors conducted a detailed evaluation of optimization methods, which organized scheduling models according to their objectives, decision variables, and algorithms. The authors observed that most research studies continue to use basic deterministic models [3]. The authors conducted a systematic review of web-based appointment systems, which analyzed 36 studies across 21 platforms to determine their impact on patient flexibility and satisfaction, but they identified privacy risks, cost issues, and user resistance to digital systems [4]. The researchers studied 98,000 ophthalmology appointments to prove online scheduling decreased private clinic no-shows from 5.9% to 1.8% but failed to decrease hospital no-shows from 11.2% to 14.3%, which demonstrated that system effectiveness depends on specific healthcare settings [5]. The research developed an optimization system that combined walk-in patient requirements with appointment patient requirements through prospect-theory-based utility optimization. The system decreased patient delays and created more equitable healthcare access [6]. A meta-narrative review of scheduling applications with AI/ML technology analyzed 11 studies, which demonstrated that these systems decreased patient absences and scheduling performance, but most research existed at the experimental level with inconsistent data quality [7]. The sensor-based patient classification system used clustering to analyze real-time vital signs for nurse scheduling optimization but encountered problems with data verification and patient information protection [8]. The two-stage stochastic optimization system developed a scheduling system that united patient scheduling with multiple physician scheduling to decrease waiting times and mismatch expenses by 51% [9]. The flexible scheduling policy for surgical residents showed positive effects on resident attendance for personal medical care and their overall health, but its effectiveness was restricted by its limited scope to one institution and a small participant number [10]. A study in Iran conducted at the Shaheed Rajaei Medical and Research Center proposed an integrated two-stage appointment scheduling model using machine learning with mathematical programming to increase the efficiency of outpatient scheduling. Patients were clustered into clinical priority using algorithms such as K-means, hierarchical clustering, DBSCAN, and OPTICS. Of the four algorithms, DBSCAN achieved the highest accuracy for Silhouette score (0.92).

A Markov Decision Process was used to dynamically allocate appointments within the model, thus allowing a reduction in waiting time for high-priority patients [11]. Likewise, another study for U.S. Veterans Affairs primary care clinics proposed a two-stage stochastic programming model combined with simulation to balance indirect waiting time (before the appointment date) with direct waiting time (in-clinic delay). Patient no-shows, cancellations, and provider availability were considered, and the model showed significant improvement in scheduling efficiency and patient satisfaction compared to heuristic methods [12]. In home healthcare, a study introduced the Vehicle Routing and Appointment Scheduling Problem to jointly optimize caregiver routing and appointment scheduling. Comparing deterministic and stochastic models, research used a VNS heuristic that showed particularly good efficiency for medium-to-large problem instances, reducing travel and overtime costs [13] Another study presented an approach of GPDR to learn interpretable appointment scheduling rules. The proposed method integrated a dimension repair process formed as an MILP model. It guaranteed consistency and achieved far better performance compared with standard genetic programming techniques in a simulated clinical environment [14]. Also, the robust optimization model was suggested for ambulatory care centers in order to minimize the operational cost and simultaneously improve the quality of service under uncertainty in service durations and patient no-shows. This model was formulated as an MILP and gave better results than the methods of deterministic optimization. It gave managerial insights related to resource allocation and operational efficiency [15]. A smart scheduling model was also developed, integrating machine learning with integer linear optimization to maximize resource utilization through overbooking strategies. By predicting attendance probabilities using Support Vector Machines, the system increased occupancy rates and utilized slots in accordance with real data from 10,000 hospital appointments [16]. In the expansion of real-time applications, this study used an AI-driven no-show prediction model on a live dashboard system in primary healthcare centers throughout the United Arab Emirates. The result was a 50.7% drop in no-shows and a reduced average waiting time by 5.7 min, pointing out that combining predictive analytics with operational systems was fundamentally important [17]. A comparison among static and dynamic scheduling approaches through stochastic mixed-integer programming and Markov Decision Processes (MDP), developed at Jeroen Bosch Hospital, Netherlands, showed the significant improvement of the latter method concerning patient access times and resource utilization, even when only a small flexible capacity allocation (~2%) was available [18]. In another paper, the researchers proposed a Graph-based Two-Agent Deep Reinforcement Learning framework, GTARS-DRL for optimizing outpatient scheduling by coupling graph neural networks with reinforcement learning. It demonstrates superior performance compared to traditional scheduling heuristics and genetic algorithms, while computational efficiency and scalability is maintained [19]. In another study, predictive models for missed appointments were developed using logistic regression, and Random Forest and XGBoost had the best performance on over a million patient visits, with an AUC of 0.92, outlining the possible use of data-driven predictions to improve health equity by reducing disparities in accessing health services [20].

## 3. Methodology

**Figure 1:**
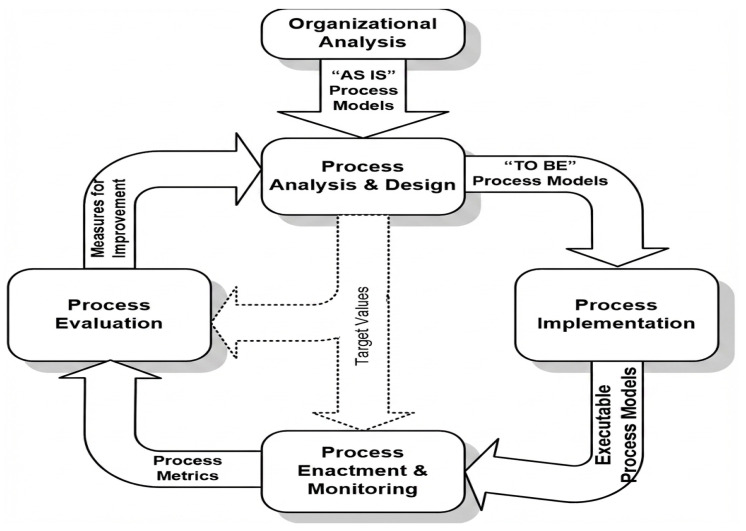
Business Process Analysis methodology.

The proposed intelligent patient appointment (IPAS) system was developed using the Business Process Analysis (BPA) methodology, as we see in Figure 1, to identify problems and redesign the appointment workflow. The current hospital appointment system (As-Is) underwent a Bizagi Modeler analysis to identify its operational weaknesses, including extended wait times and unnecessary human intervention. The analysis results led to the creation of a new (To-Be) model, which uses artificial intelligence (AI), machine learning (ML), and natural language processing (NLP) to perform automated patient assessment and appointment booking.

The baseline models evaluated were as follows:

- Random Forest on structured features only (demographics, vitals, and basic symptoms);
- Standard BERT classifier (BERT-base) fine-tuned on the same text inputs.
- Optionally, a simpler BiLSTM with randomly initialized embeddings.

BioBERT is pre-trained on biomedical corpora, which better captures clinical terminology and abbreviations than standard BERT, improving the representation of free-text symptom descriptions and triage notes. The BiLSTM layer on top of BioBERT embeddings allows the model to capture temporal and contextual dependencies within the patient’s narrative (e.g., evolution of symptoms, modifiers like “sudden”, “worsening”), which tree-based models (Random Forest) and plain BERT classifiers may not exploit as effectively. Multi-modal extensibility is an architecture that can naturally integrate additional tokenized inputs (e.g., structured codes and image-derived labels) into the sequence, aligning with our description that “IPAS analyzes patient input, combined with historical medical data and laboratory results, to generate an initial clinical assessment.” BioBERT + BiLSTM was selected because it consistently outperformed the baselines on the same dataset and aligns with the clinical text-heavy nature of triage. The Bizagi simulation tool processed both models to determine their operational performance through time measurements, resource consumption, and system congestion points before deployment. The assessment of process quality, error reduction, and continuous improvement used SWOT analysis together with Six Sigma and Total Quality Management (TQM) techniques. The evaluation of As-Is and To-Be models helped determine the extent of improvement in waiting time, scheduling accuracy, operational efficiency, and Key Performance Indicators (KPIs). To ensure patient safety in cases where the AI triage model may generate false-positive or false-negative classifications, the IPAS framework incorporates a multilayered safety and escalation protocol. All triage outputs pass through a rule-based clinical safety layer that detects “red-flag” symptoms—such as chest pain, neurological deficits, severe trauma, or rapidly worsening conditions—and automatically overrides any low-priority prediction. In such cases, the system immediately directs the patient to the nearest emergency department and transmits the chatbot’s assessment to the receiving facility, consistent with the system’s design that “in emergency situations, the IPAS has the ability to determine urgent cases and send the patient’s information instantly” (document excerpt). Additionally, all high-urgency classifications are routed to a human clinician for rapid verification before final disposition. This hybrid human–AI oversight model minimizes the risk of under-triage and establishes a controlled safety mechanism for continuous monitoring of triage accuracy during deployment.

### 3.1. Design

**Figure 2:**
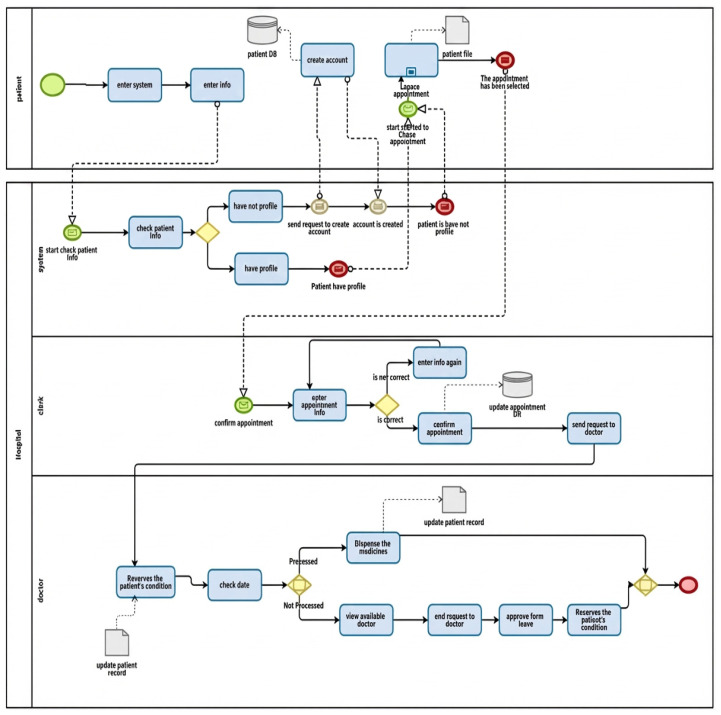
As-Is process flow for patient appointment scheduling.

This section presents process designs that are represented through Figure 2. The “As-Is” model depicts the present status of the workflow in terms of its main components, drawbacks, and inefficiencies.

#### As-Is Model

The process begins when the patient wishes to schedule an online appointment. Then, the patient enters information. The system tests whether the patient has an account or not. If they do not have an account, they are asked to enter their information. Otherwise, the process is completed. The patient chooses an appointment. The system checks for availability. If none are available, the patient is asked to select an alternative appointment. Otherwise, the appointment is booked, and a confirmation message is sent. The receptionist clerk checks if the patient has an appointment. If an appointment exists, the appointment status is updated and then sent to the doctor. The doctor evaluates the patient’s condition. If the patient is treated, the medication is dispensed, and the operation is completed. Otherwise, the patient is sent to another specialist doctor.

### 3.2. Analysis

This section covers a detailed analysis of the current system using different quality and strategic assessment tools, such as Total Quality Management, SWOT analysis, and the Six Sigma methodology. The objective of this analysis is to reveal the system’s strong points, weaknesses, opportunities, and threats while assessing process efficiency, consistency, and quality of performance. By integrating these various approaches, the research intends to determine the root causes of existing problems, provide a clear definition of areas for improvement, and lay a solid foundation for the design of an optimum and sustainable future model.

#### SWOT Analysis

Strengths:

- The system combines AI with machine learning (ML) and NLP to perform automated triage and appointment scheduling, which enhances medical diagnosis precision and decreases human involvement in administrative tasks.
- The system allows patients to book appointments instantly while it determines their treatment priority to reduce their waiting time and maximize doctor availability.
- The system uses data analytics, which incorporates medical history and laboratory results to enhance treatment precision.
- The Bizagi simulation system, together with BPMN modeling, enables organizations to obtain quantifiable performance data before deploying their solution.

Weaknesses:

- The system needs access to high-quality medical data, which exists in a single format, because different hospital environments with diverse systems reduce model precision.
- The process of system integration with existing hospital systems leads to delays during the initial deployment phase.
- The use of AI models in clinical decision support systems creates problems with explaining and making system decisions transparent to users.
- The system faces challenges in user acceptance because older patients and those who lack digital skills may struggle to use the system.

Opportunities:

- The system has the potential to link with IoT devices and Electronic Health Records (EHRs) for scheduling appointments based on continuous patient monitoring.
- The system uses cloud-based infrastructure to expand its reach across healthcare networks and remote clinics.
- The system implements predictive and prescriptive analytics to support emergency triage and dynamic resource allocation.
- The system benefits from increased digital transformation interest in healthcare worldwide, which boosts its chances of market adoption.

Threats:

- Healthcare organizations face two main security threats because of GDPR and HIPAA regulations, which protect patient data privacy.
- Medical staff members show resistance to workflow modifications because they worry about automation taking over their work.
- The high expenses for system deployment and upkeep create challenges for hospitals with limited resources.
- The system faces a risk of developing discriminatory algorithms which could lead to unfair treatment of patients during scheduling and medical service delivery.

### 3.3. Total Quality Management (TQM)

The IPAS framework implements Total Quality Management (TQM) to achieve continuous improvement, patient-centered care, and operational excellence throughout the system development and deployment stages. The following dimensions of TQM principles were used for implementation.

#### 3.3.1. Customer Focus

The IPAS system delivers patient satisfaction through its AI and NLP-based assessment system, which reduces waiting times, enhances scheduling precision, and provides individualized triage services. The system collects feedback from patients and healthcare staff members to make continuous design enhancements.

#### 3.3.2. Continuous Improvement

The project implements Plan–Do–Check–Act (PDCA) cycles to enhance workflows, which were discovered through Business Process Analysis (BPA) and Bizagi simulation activities. The system tracks performance metrics, including error rates, waiting times, and scheduling efficiency, to detect areas for improvement.

#### 3.3.3. Leadership and Collaboration

The hospital administrators, together with IT personnel and clinical specialists, worked together to create new processes for the system. The collaborative approach between stakeholders led to better quality results and easier system adoption of the new scheduling framework.

#### 3.3.4. Process Management

The implementation of BPMN modeling together with Six Sigma DMAIC standards created standardized workflows that decreased operational variability and achieved departmental reliability. The system established quality targets for all process stages to achieve transparent results that could be measured.

#### 3.3.5. Employee Involvement and Training

The TQM approach provided training to medical and administrative staff for system operation, data analysis, and quality performance tracking, which developed an environment of responsible and efficient work practices.

The TQM principles strengthened IPAS to provide dependable, high-quality healthcare services that focus on patient needs through process optimization between people, systems, and technology systems.

##### Six Sigma

The Six Sigma methodology proved to be effective in improving process quality and reducing errors and execution time, thus positively impacting the general performance efficiency and customer satisfaction. With the five phases of the methodology, namely, identify–measure–analyze–improve–control, root causes were identified with practical solutions, thus contributing to improvement in workflows and measurable results. It shows that the Six Sigma methodology can be applied to bring in continuous improvement and ensure process quality across various sectors.

##### Pareto Chart

**Figure 3:**
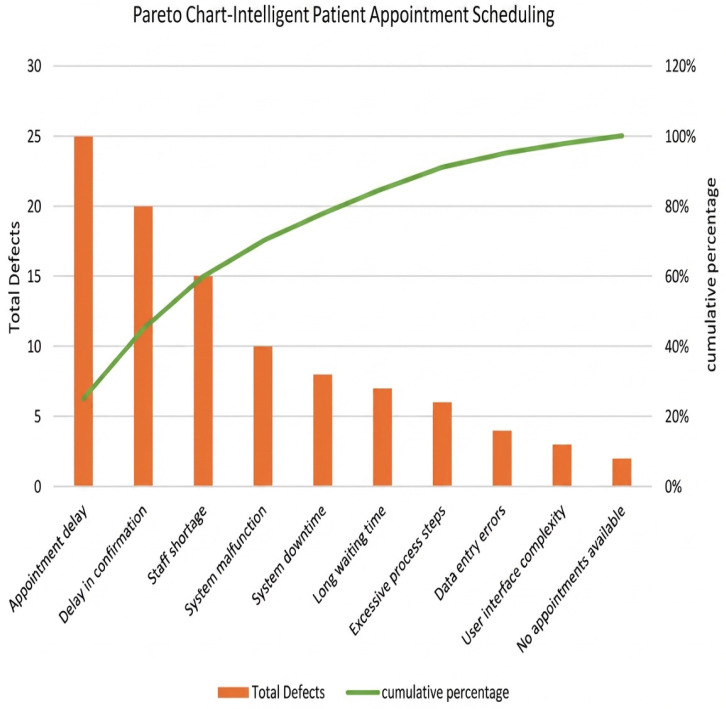
Pareto chart analysis.

The current system (As-Is) shows that the booking cycle takes 155 min (2.5 h). Steps that increase waiting time include manual data entry, appointment confirmation by staff, and notification to the doctor. By using a Pareto chart as in Figure 3, we identify the most common causes of delays.

##### Fishbone

**Figure 4:**
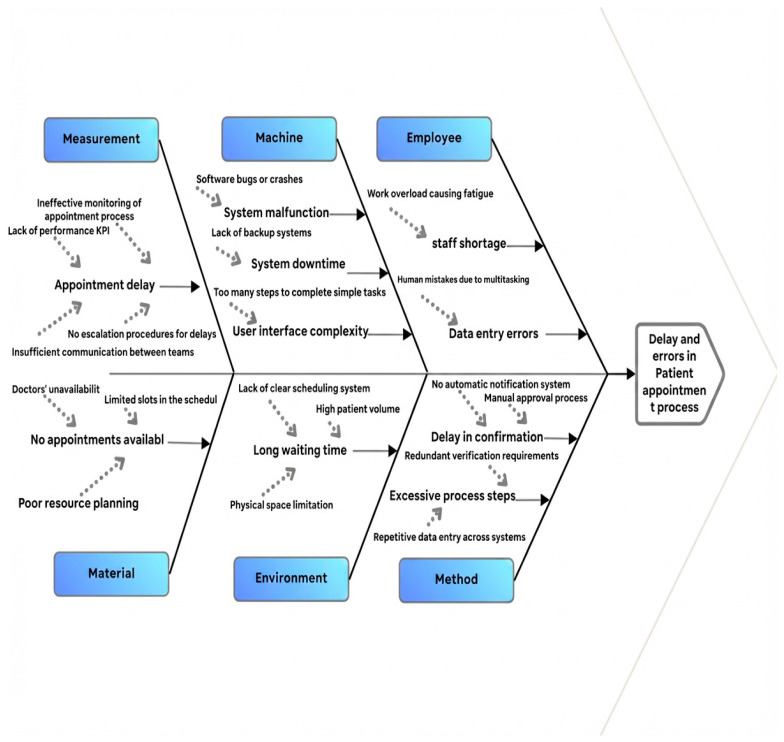
Fishbone analysis.

The Fishbone diagram shown in Figure 4 reveals several factors contributing to delays and increased waiting times.

### 3.4. KPI Chart

**Figure 5:**
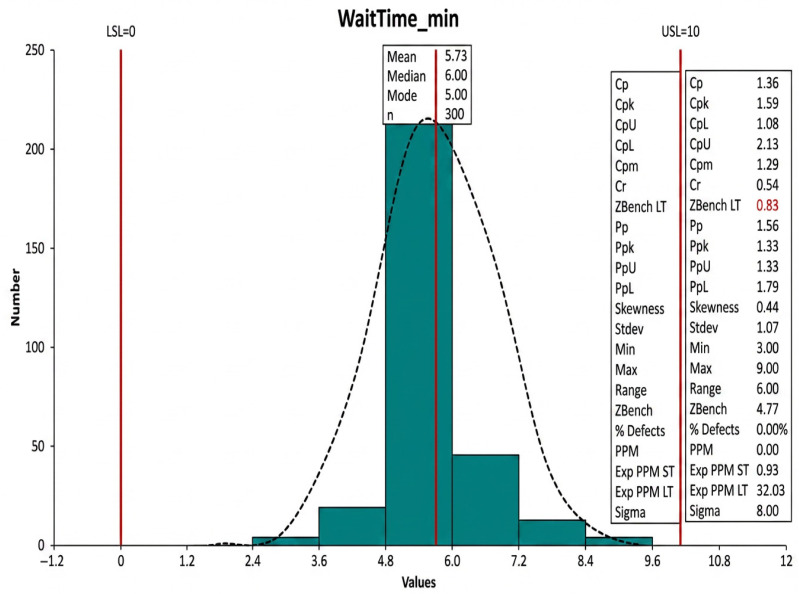
Process capability for waiting time.

- Development of an intelligent chatbot linked to the hospital’s database (Figure 5).
- It analyzes symptoms submitted by the patient (text or image).
- It categorizes the condition (dermatological, orthopedic, psychological, etc.) and instantly displays the appropriate doctors.
- It automatically books appointments without human intervention.
- It sends alerts to both the doctor and the patient.

**Figure 6:**
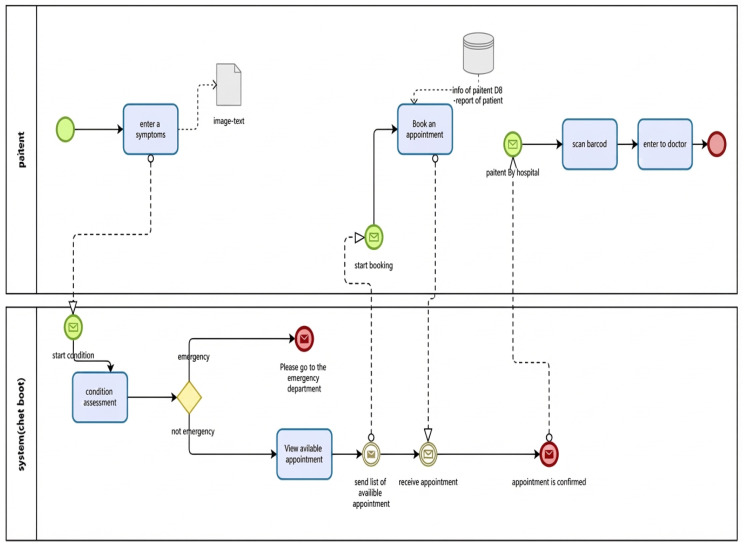
To-Be process flow for patient appointment scheduling.

Results after improvement:

- Booking time reduced from 155 min to just 5.73 min, as shown in Figure 6.
- Human intervention reduced by 70%, as shown in Table 1.
- Increased system efficiency and service speed.

### 3.5. V. Redesign

The Figure 6 “To-Be” design defines an enhanced future state of the process, which has been developed based on an analysis of current challenges and desired performance outcomes. This comparison of both models brings out the basis for enhancement that will be accomplished by the proposed design in optimizing efficiency, reducing errors, and improving overall system performance. A chatbot will be implemented, linked to the hospital’s database or the patient’s medical records service. The patient then sends a written or photographic description of the symptoms, which are analyzed by algorithms that first classify the condition (dermatological, orthopedic, psychological, etc.). Appropriate doctors are then displayed, and the patient books an appointment with the specialist. If the condition is critical, the patient will be directed to the nearest emergency department. The system then sends the symptom data and the chatbot’s report to the patient’s file upon arrival at the doctor’s office.

## 4. Result

The IPAS prototype was deployed in a simulated hospital environment using a Bizagi automation server connected to a mock EHR database (n = 5000 synthetic patient records). The triage classifier (BioBERT + BiLSTM) achieved a **test AUC 0.92** and an **F1-score** of **0.87** across five urgency levels and 12 specialties, validated on held-out PhysioNet ED data. The results obtained after analysis and implementation proved that improvement in operational efficiency, reduced booking time, and enhanced quality of the healthcare services are indeed achieved with the development of the Intelligent Appointment System. Quantitative analysis, using tools such as the Six Sigma methodology, allowed the root causes of delays to be precisely identified, thereby reducing the average booking time from 2.5 h to less than one hour while also reducing human intervention by 70%. During the Business Process Analysis, the As-Is workflow was found to require a total booking cycle of **155 min (2.5 h)**, primarily due to manual data entry, staff-based confirmation steps, and sequential communication delays, as noted in the analysis that *“the booking cycle takes 155 min (2.5 h)”* (document excerpt). To maintain consistency across the methodology and results, this value replaces the earlier typographical reference to “1 h” in Table 1. The corrected baseline ensures alignment with the Pareto analysis and KPI evaluation, where the To-Be model demonstrates a reduction from 155 min to **5.73 min**, reflecting the substantial efficiency gains achieved through automation and AI-driven scheduling. In addition, SWOT analysis was used to guide this improvement process based on the technical strengths and integration and community acceptance weaknesses of the system, in addition to opportunities provided by digital transformation within the healthcare sector and risks regarding security and privacy threats. The integration of Total Quality Management principles achieved an excellent balance between technological and human factors, thus leading to increased patient satisfaction and more efficient and sustainable institutional performance.

## 5. Conclusions

The IPAS framework has turned out to be effective in enhancing the efficiency of hospitals by saving time in booking and communicating between patients and doctors. The system has managed to speed up administrative procedures and accelerate service delivery, benefiting both patient satisfaction and the overall quality of the healthcare experience. The project indicated that effective utilization of modern technologies helps in developing administrative and medical systems for accurate, smooth, and sustainable performance. The achievement is a step toward digital transformation in the health sector and contributes to its objectives of raising the efficiency and quality of services provided for beneficiaries. One of the limitations of this work is that the reported improvement rates were derived from Bizagi simulation rather than real-world clinical deployment. which reflects a process-model evaluation rather than validated clinical outcomes.

## 6. Future Work

The IPAS framework will receive future development through clinical testing, system expansion, and hospital information system real-time connection. The appointment allocation process will achieve better results through predictive analytics and reinforcement learning, which will use patient urgency data, resource status, and past treatment information. The research team will study federated learning as a data privacy solution to fulfill healthcare rules while preserving model accuracy. The Bizagi simulation models will receive additional data for performance testing under different operational conditions and unpredictable situations. The research will evaluate KPI performance between AI-based scheduling systems and conventional scheduling approaches through experimental studies. The research will explore how to link IoT-based patient tracking systems to create flexible scheduling systems that adapt to changing healthcare settings. Risks associated with privacy and compliance concerns will be addressed in future system development by considering technical mitigation strategies, including stronger data protection measures and privacy-preserving approaches.

## Figures and Tables

**Table 1:** Impact of improvement.

Index	Before Improvement	After Improvement	Improvement Ratio
Full booking time	1 h	5.73 min	80%
Human intervention	High	Very limited	64%
Patient satisfaction	Middle	High	+60%

## Data Availability

The original contributions presented in this study are included in the article. Further inquiries can be directed to the corresponding author.
